# News and Coming Events

**Published:** 1907-11-09

**Authors:** 


### NEWS AND COMING EVENTS.

Alvarenga Prize.—The College of Physicians of Philadelphia announces that the Alvarenga Prize for 1907 has been awarded to Dr. William Louis Chapman, Providence, R.I., for his essay entitled "Post-operative Phlebitis, Thrombosis, and Embolism." The next award of the prize, amounting to about $180, will be made on July 14, 1908. Particulars may be obtained from Dr. Thomas R. Neilson, Secretary of the College of Physicians of Philadelphia.

The risk of fire at our large asylums is an ever present one, and many are protected by their own appliances, a large number having steam fire-engines, either in a fixed or portable form. The latest building to be thus protected is the new Dykebar Asylum, which has been erected by the Renfrew District Lunacy Board. The fire engine installed here is of stationary form, and is a powerful pump of Merryweather & Sons' double vertical "Greenwich Gem" pattern, similar to one they supplied a few years ago to the London, Brighton, and South Coast Railway for the protection of Newhaven Harbour.

A general practitioner, in a recent letter to the *Manchester Guardian*, expresses himself strongly as to the niggardly pay of institutional medical officers. "The fact is," he says, "many hospitals employ sweated labour. . . . The average rate of remuneration is £90 per annum, with board and lodging. This is much below the standard rate of pay in the profession, and is not a 'living wage.' I would not spend £1,000 for the education of a son of mine to enable him to earn £90 per annum. Far better make him a bricklayer. He might then perhaps live in comfort on such a wage."

Princess Victoria of Schleswig-Holstein, Lady President of the League of Mercy for the counties of Berkshire, Buckinghamshire, Hampshire, and Oxfordshire, was present at a drawing-room meeting held on October 31 at Nuneham Park, Oxford. A report on the organisation of the League in the four counties during the presidency of Princess Victoria was read by Mrs. Murdoch, who mentioned that since Her Highness took up the work of the League in 1903 the collections in the four counties had risen from about £18 to £1,719. Sir Henry Burdett, Hon. Secretary of the League, delivered an address showing the benefit of its work for local hospitals. Taking ten of the Home Counties in which the League of Mercy was at present at work, the contributions last year to the hospitals through the League amounted to no less than £6,900.

Arrangements are being made at Queen Charlotte's Hospital whereby it will shortly be possible to admit fourteen medical students per month (168 per annum) to the practice of the hospital in order to receive preliminary instruction in practical midwifery in accordance with the recommendations of the General Medical Council. This instruction will include : (1) Practical instruction in the methods of examination of pregnant women; (2) delivery of women in labour under the direct supervision of a medical officer of the hospital; (3) practical instruction in the treatment of the mother and child during the puerperium, including clinics held four times weekly by the visiting medical staff; (4) instruction in the clinical laboratory of the hospital. Students will be accommodated in the residential college adjoining the hospital. The inclusive fee for hospital practice and board and lodging will be £12 12s. per calendar month. 1,704 women were delivered in the hospital last year (1906), of which nearly two-thirds were primiparæ. A large number of the cases were abnormal.

Dr. John H. Dauber has been elected President of the Chelsea Clinical Society.

The Goldsmiths' Company has sent a donation of 100*l*. to Queen Charlotte's Lying-in Hospital.

The Treasurer of the London Homœopathic Hospital, Lord Cawdor, will preside at a Festival Dinner on behalf of the Extension Fund on November 20 next at the Hôtel Ritz, and we earnestly invite the assistance of the charitable public in the effort which the Board of Management and the medical staff are making to complete the fund of £30,000 by Christmas next.

The scheme for the extension of the Salford Royal Hospital provides for the purchase of land adjoining the existing block of buildings, for an increase in the number of beds from 135 to 200, accommodation for the nurses and staff, and alterations in the out-patient department. Up to date contributions and promises to the amount of £27,081 have been received, but as the estimated cost of the extensions and alterations totals £70,000, the Special Committee has issued an urgent appeal to the general public for further funds. The hospital is the only general hospital in Salford, and ministers to a population of over 266,000, and the present available accommodation is, therefore, totally inadequate. For the last ten years there has been a deficiency in the income of the institution, necessitating the realisation of certain of the capital funds of the charity in order to meet recurring debts.

